# Multifocal tuberculosis revealed by a sternal swelling in an immunocompetent child

**DOI:** 10.1099/acmi.0.000795.v3

**Published:** 2024-08-05

**Authors:** Ghizlane Chehrastane, Elmostafa Benaissa, Abdelilah Radi, Amal EL Hassani, Mostafa Elouennass

**Affiliations:** 1 Department of Microbiology, Mohammed V Military Instruction Hospital, Rabat, Morocco; 2 Department of Pediatrics and Neonatology, Mohammed V Military Instruction Hospital, Rabat, Morocco

**Keywords:** multifocal tuberculosis, real-time PCR, sternal swelling

## Abstract

Tuberculosis (TB) is one of the most common pathogens of bacterial lung infections, especially in underdeveloped nations like Morocco, where the incidence of TB was 97 cases per 100 000 persons in 2019. Thanks to its national TB prevention and control plan, Morocco was able to achieve remarkable progress in the management of TB with an 80% reduction in the total number of patients diagnosed with TB between 1980 and 2018. The national plan also allowed us to reach and maintain a therapeutic rate above 86% since 2002. Sternal TB is a rare clinical condition accounting for 1% of all musculoskeletal TB cases. Due to its rarity and the lack of awareness of clinical presentations, the diagnosis of sternal TB can be quite complex. We describe the case of a 14-year-old Moroccan patient consulting in the Military Hospital Mohammed V-Rabat with central chest pain for 4 months which was not associated with breathing, physical exercise or eating. The patient also had a history of asthenia, fever and weight loss. A computed tomography scan of the chest showed a destructive lesion of the sternum. Afterward, a chirurgical biopsy was performed and enabled to confirm the microbiological diagnosis of TB with the realization of the real-time PCR. The antitubercular therapy was given to the patient who had complete resolution of symptoms. This condition should be included in the differential diagnosis of chronic chest pain that mimics costochondritis particularly in patients from endemic areas.

## Data summary

All data associated with this work are reported within the article.

## Introduction

Tuberculosis (TB) of the bone is the result of hematogenous dissemination of bacilli following primary infection. TB of the sternum is an exceedingly rare manifestation of extrapulmonary TB, representing less than 2% of all osteomyelitis [1] and 2–3% of osteoarticular TB [2]. The diagnostic and medical management of patients with sternal TB is challenging because of its rarity and the lack of awareness, as well as the clinical presentation of lesions with nonspecific signs and symptoms that may suggest chronic osteomyelitis, malignancy and other similar disorders [3,4], hence the importance of microbiological diagnosis offering greater sensitivity than medical imaging technology and the tuberculin skin test.

## Objective

We report a case of multifocal TB in an immunocompetent child.

## Case presentation

**Fig. 1.**
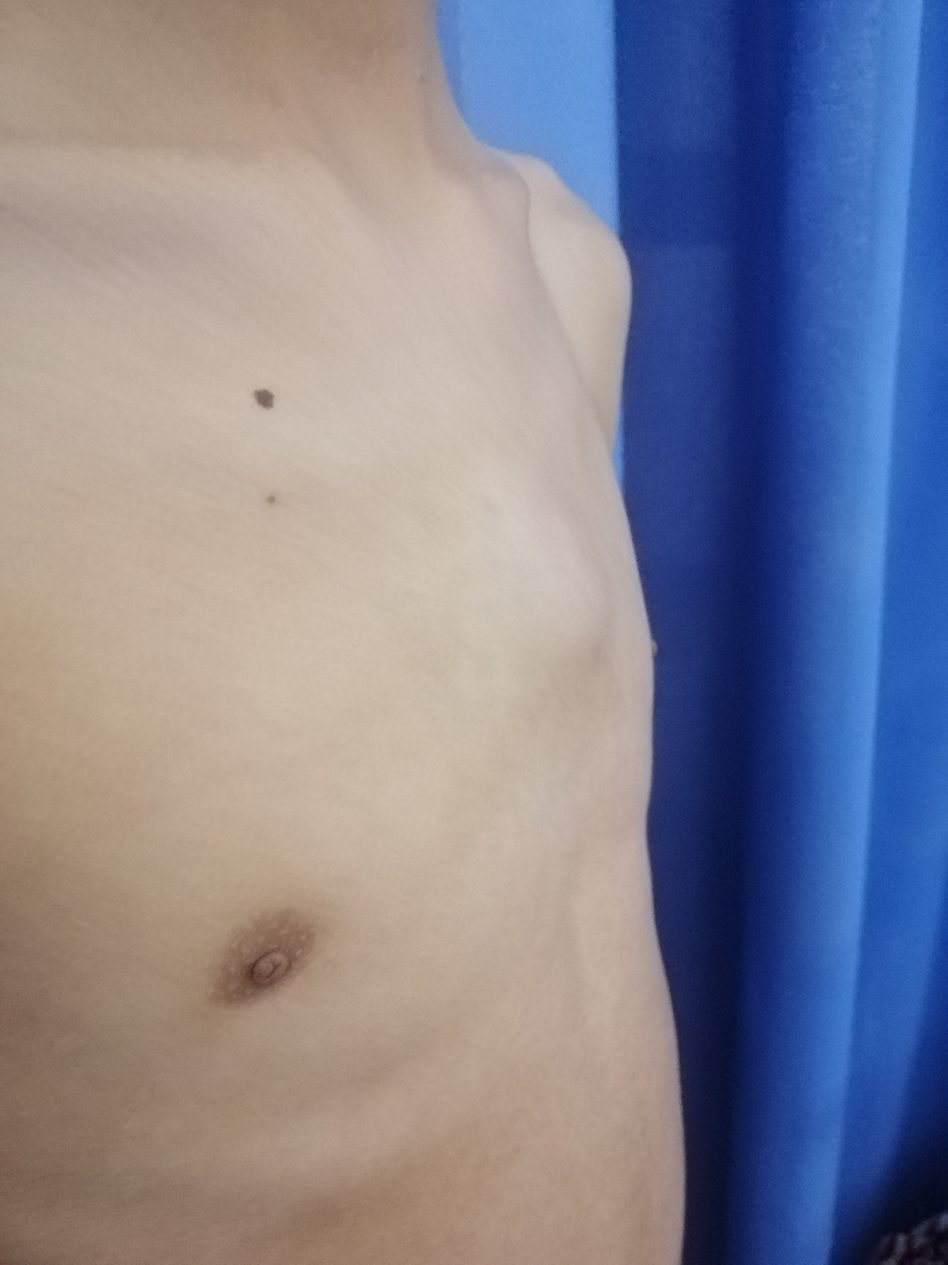
Lateral view of the sternal swelling.

The patient was 14 years old and had been vaccinated with bacille Calmette–Guerin and with a previous episode of TB exposure. The history of the symptoms dates back to 4 months with the installation of a chest pain with a burning sensation radiating towards the shoulders, followed by the appearance after 1 month of a palpable swelling of about 4 cm in size at the level of the upper part of the sternum evolving in a context of apyrexia, asthenia with night perspiration and a weight loss of 3 kg (Fig. 1).

**Fig. 2.**
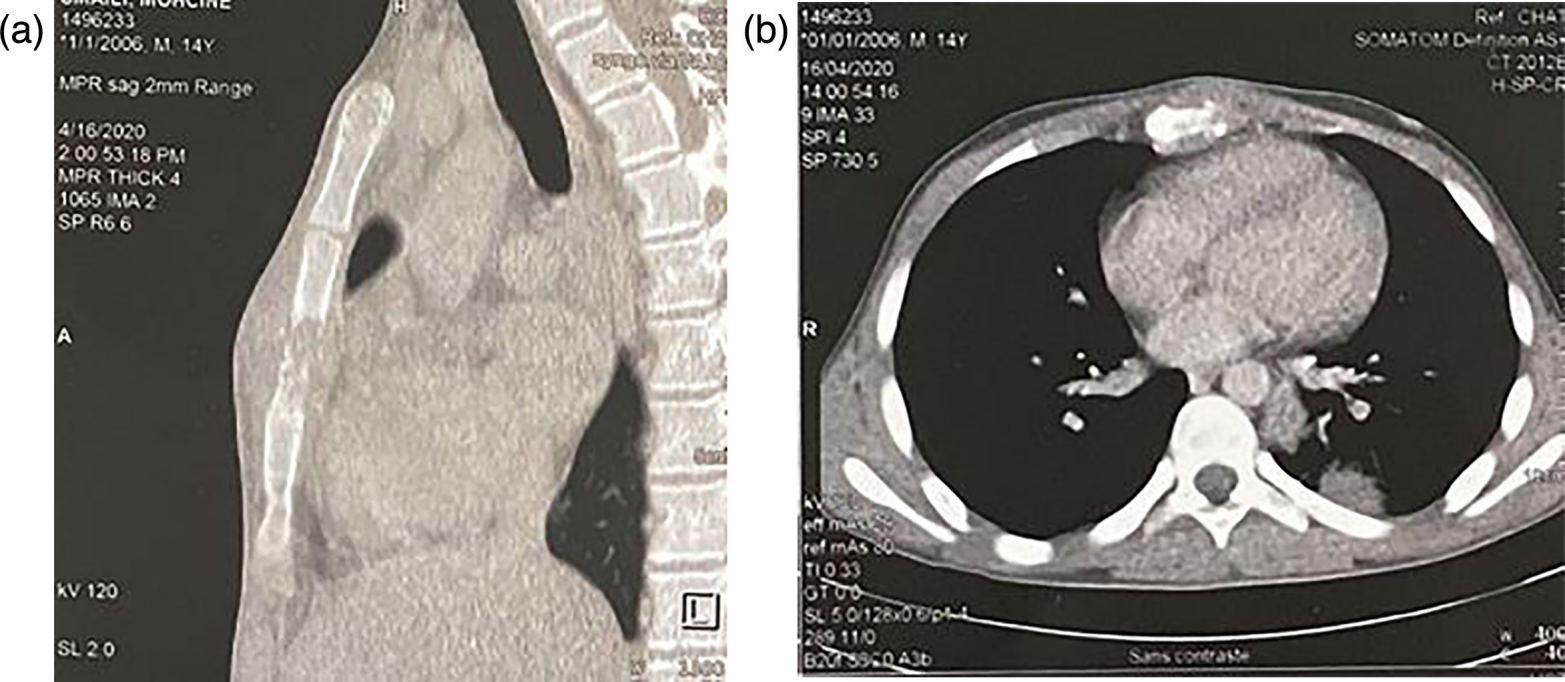
Chest CT scan showing an osteolytic tissue mass centred on the sternum (left, axial section; right, sagittal section).

The chest X-ray showed a systematized heterogeneous opacity on the left medio-thoracic side. The computed tomography (CT) scan showed an osseous lysis of the sternal body with infiltration of the soft tissues, a systematized pulmonary condensation on the left side associated with medio-thoracic mediastinal adenopathies and a splenomegaly with a hypodense intra-splenic image (Fig. 2). Further clinical examination was unremarkable.

The initial biological tests showed hyperleukocytosis (white blood cells=17.6 Giga/L) with a predominance of neutrophils (7.9 Giga/L), microcytic hypochromic anaemia (haemoglobin=10.9 G dL^−1^) and thrombocytosis (platelets=500 Giga/L). The C-reactive protein was 85.1 mg l^−1^ associated with a disturbance of the hepatic balance owing to hepatic cytolysis with ALAT=112 IU/L and ASAT=120 IU/L.

Bacteriological examination of the surgical sternal biopsy and respiratory specimens did not show *acid-fast* bacillus on direct examination by Auramine and Ziehl–Neelsen staining, whereas real-time PCR performed with the GeneXpert-Cepheid automated system detected the presence of *Mycobacterium tuberculosis* complex at very low level without detection of rifampin resistance genes. The GeneXpert is a rapid test that can simultaneously identify *M. tuberculosis* and detect the resistance to rifampin from sputum. It is a nucleic acid amplification method designed as an integrated DNA extraction and real-time PCR. A reagent is added to a concentrated sputum pellet to liquefy the specimen and kill any mycobacteria within. The cartridge is loaded into the GeneXpert Analyzer, and the results are delivered in less than 2 h.

**Fig. 3.**
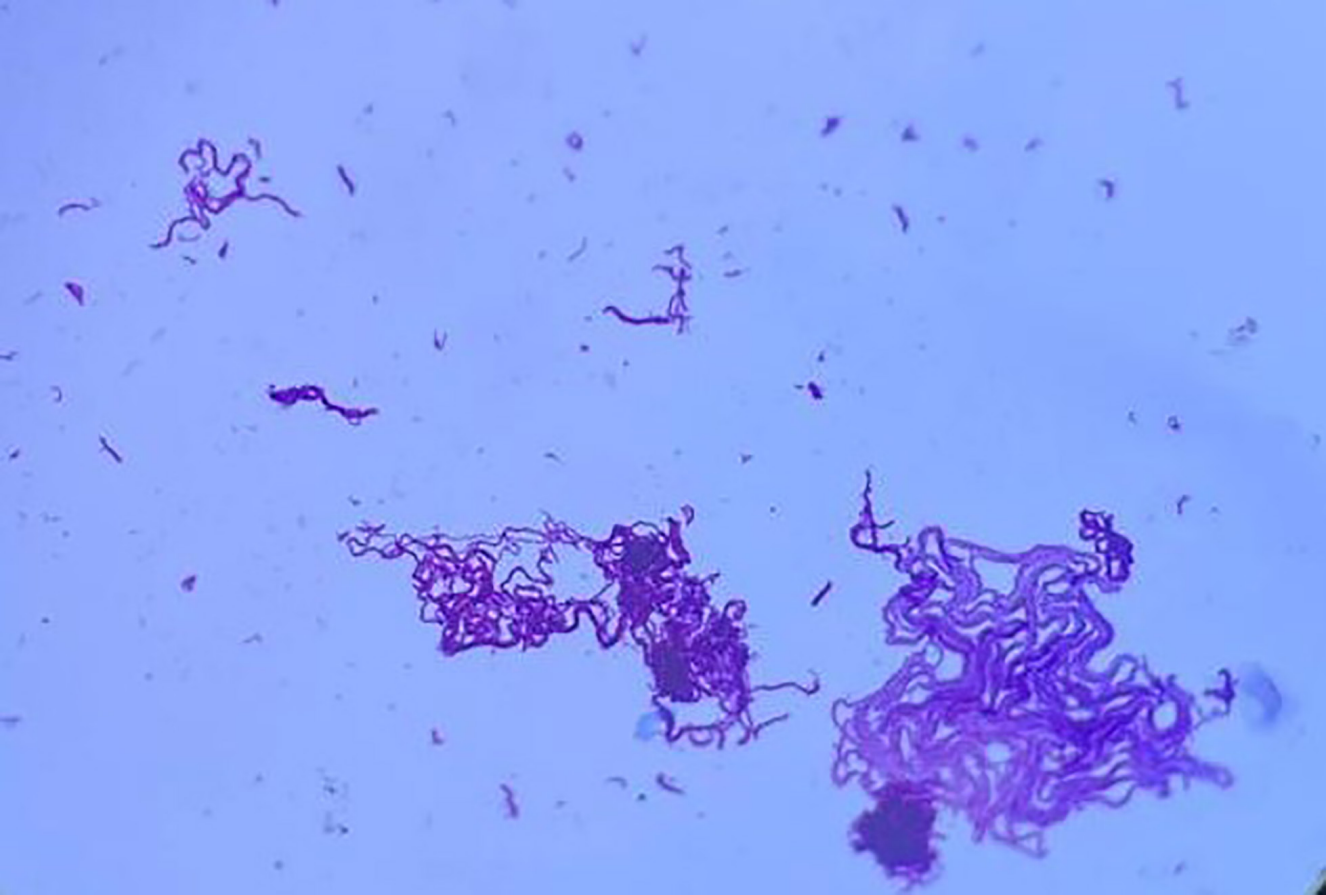
The appearance of bacilli after Ziehl–Neelsen staining from colonies of liquid medium MGIT.

**Fig. 4.**
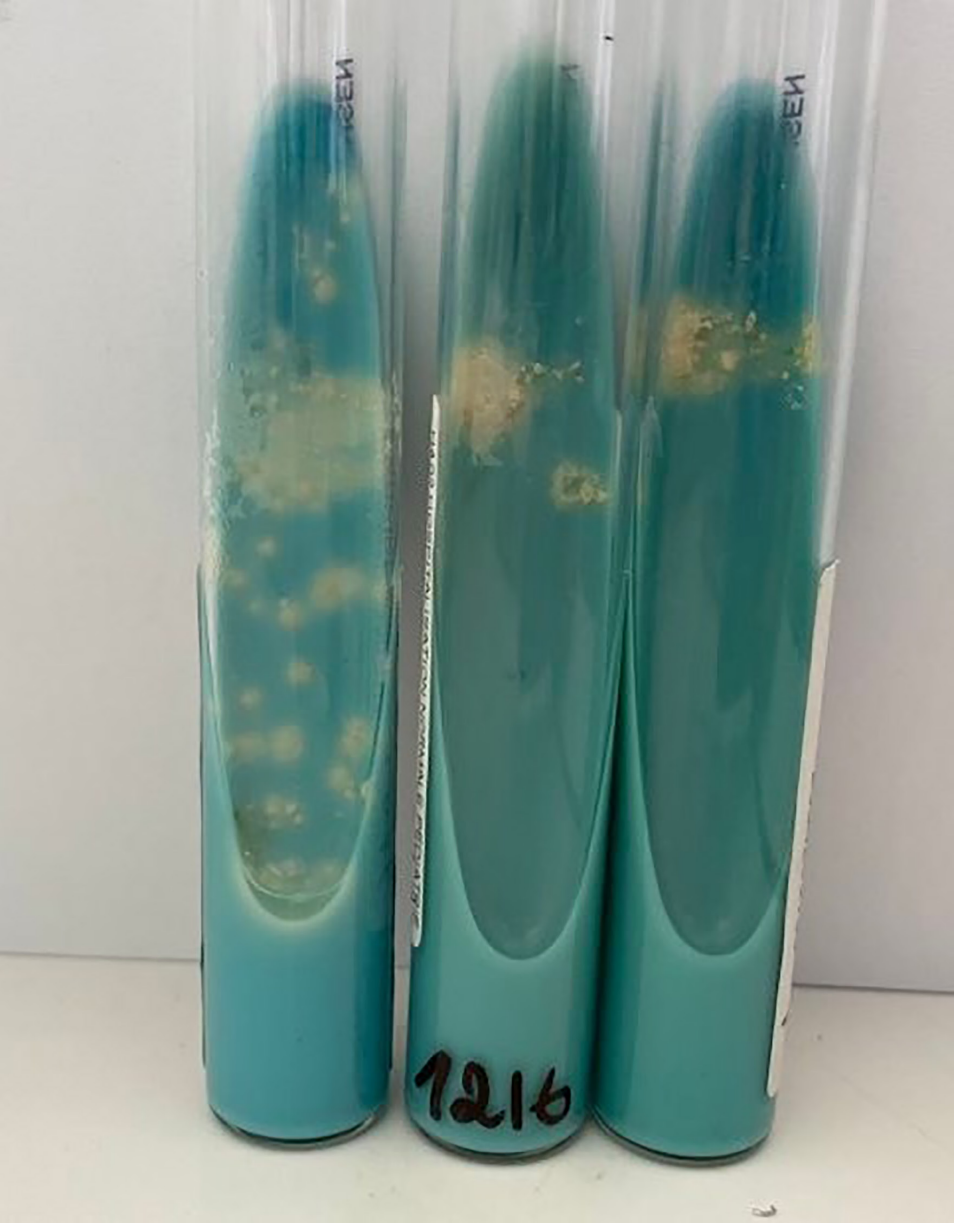
LJ medium showing coloured colonies of 3–4 mm diameter with a cauliflower-like appearance and adherent to the medium.

The culture of the biopsy was positive after 6 days on the liquid medium MGIT (mycobacterial growth indicator tube containing 7 ml of modified Middlebrook 7H9 Broth base) (Fig. 3) and 19 days on the solid medium LJ (Lowenstein–Jensen (Fig. 4)).

In the light of the bacteriological and radiological results, multifocal TB was retained as the diagnosis, and an antitubercular medication was initiated as indicated by the national TB prevention and control programme (PNLAT) with the use of rifampin, isoniazid, pyrazinamide and ethambutol under the protocol 2 (RHZE)/10 (RH).

During his hospitalization, the patient presented intermittent back pain of mechanical character without limitation of the vertebral joints or impotence with a completely normal neurological examination. A CT scan of the cervical–dorsal–lumbar spine was performed and revealed multifocal spinal bone affection involving the spinous process of C7. A transverse process and posterior arch of D6 filling the right foramen and the left costo-vertebral joint of D11 were also revealed. The clinicobiological improvement was marked by a progressive resolution of the swelling, an apyretic state with weight gain and a decrease in hepatic cytolysis.

## Discussion

TB remains an important global cause of morbidity and mortality, particularly in developing regions. In Morocco, 29 327 patients were diagnosed with TB and put on antitubercular drugs in 2021 within the framework of the PNLAT, with an estimated 34% decrease in the incidence of the disease and a 68% decrease in mortality over the last 30 years, with therapeutic success rates maintained at over 85% [5].

TB infection of flat bone remains rare even in endemic countries, and sternal localization has an incidence of only 2–3% of cases of osteoarticular TB [2] and remains exceptional in children [4,5]. It occurs mostly after cardiac surgery, bacille Calmette–Guerin vaccination[6] or in association with thalassemia, millary TB and immunosuppression [7]. It can also result from direct extension from mediastinal adenopathies [8] that are supposed to be the consequence of bacillary spread by contiguity from intrathoracic lymphatics during a primary TB infection with a pulmonary origin, which seems to be the case of our patient.

A meta-analysis conducted by Shi-Min Yuan of articles published between 2000 and 2013 showed that isolated TB of the sternum was observed in 96 patients (60.4%), association of sternal TB with the invasion of peritonsillar tissue (joints, cartilage and muscles) in 32 patients (20.1 %) and a multifocal TB 31 patients (19.5 %) [9].

The occurrence of radiological signs happens after a considerable delay compared to the clinical manifestations like abscesses or sinuses that appear much before the focus is found [10]. The symptoms vary according to the progression of osteomyelitis. However, tuberculous sternal osteitis presents as a painful swelling of the anterior chest wall overlying the sternum with general signs such as fever, weight loss and anorexia.

The discovery of the disease is often made at a later stage of complications such as sternal fracture, which was not the case in our patient who only presented osseous lysis of the sternal body with minimal symptoms evolving for 4 months before hospitalization.

An increase in total white blood cells or other inflammatory markers including erythrocyte sedimentation rate and C-reactive protein are neither specific nor completely accurate, whereas chest film did not reveal any abnormalities in about 70% of patients [4].

The diagnosis of sternal TB relies in part on imaging, especially thoracic CT, which often shows a lytic lesion of the sternal body, surrounded by a tissue mass that invades the soft tissue externally and/or mediastinal structures internally [11]. In our case, the reported thoracic CT scan characterizes the extension of the bone destruction and the involvement of the soft tissues located opposite, which was manifested as a hypodense lytic process. However, the diagnosis remains challenging due to the absence of other pulmonary or extrapulmonary injuries evocative of TB, not to disregard the fact that other diseases may have the same radiological and clinical features like chronic pyogenic osteomyelitis, tumours or blood disorders [12].

Therefore, a surgical or an ultrasound-guided biopsy is required in most cases to provide a diagnosis using microbiological and histological methods when radiological findings do not allow to determine the cause of osteomyelitis especially when the lesions grossly emulate pyogenic abscess or neoplasm [12,13].

In our case, the real-time PCR, which has been reported to be used for the diagnosis of bone TB with a sensitivity of 85% and a specificity of 80% [14], was the fastest and most sensitive examination to confirm the diagnosis of sternal TB from surgical sternal biopsy and respiratory specimens.

## Conclusion

TB does not always appear with typical pulmonary manifestations in children. The possibility of sternal TB, even if it is extremely rare, must be considered in any patient presenting a sternal swelling or abscess, and the awareness of clinicians, especially in underdeveloped countries where TB is endemic, of atypical clinical presentations of this deadly infection can help improve the medical management of the patients.

## Abbreviations

CT: computed tomography
LJ: Lowenstein–Jensen
MGIT: mycobacterial growth indicator tube
PCR: polymerase chain reaction
TB: tuberculosis

## References

[1] Sachdeva R, Sachdeva S, Arora S (2013). Sternal tuberculosis. Ann Med Health Sci Res.

[2] Bhatia VY, Aggarwal V, Sharma U, Gupta A (2009). Primary tuberculous sternal osteomyelitis: a clinical rarity. Asian Cardiovasc Thorac Ann.

[3] Rajan J, Bizanti K (2021). Sternal swelling presenting as tuberculosis: a case report. *J Med Case Reports*.

[4] Asif A, Dabral L (2021). Sternal tuberculosis: case series of two cases. J Orthop Case Rep.

[5] Accueil (2022). Consulté le: 19 octobre 2022. [En ligne]. https://www.sante.gov.ma/Pages/Communiques.aspx?IDCom=408.

[6] Kato Y, Horikawa Y, Nishimura Y, Shimoda H, Shigeto E (2000). Sternal tuberculosis in a 9‐month‐old infant after BCG vaccination. Acta Paediatrica.

[7] Allali N, Dafiri R (2005). Localisation sternale de la tuberculose osseuse. J Radiol.

[8] Tristano AG, Willson ML, López A (2004). Sternal osteomyelitis caused by *Mycobacterium tuberculosis*: case report and review of the literature. Infect Dis Clin Pract.

[9] Yuan S-M (2016). Sternal mycobacterial infections. Ann Thorac Med.

[10] Anand P, Sarin N (2018). Isolated sternal tuberculosis presenting as a chest wall abscess: a case report. Iran J Med Sci.

[11] McLellan DG, Philips KB, Corbett CE, Bronze MS (2000). Sternal osteomyelitis caused by mycobacterium tuberculosis: case report and review of the literature. Am J Med Sci.

[12] Aghoutane EM, Fezzazi R (2011). Tuberculose sternale isolée chez l’enfant: à propos d’un cas. Rev Pneumol Clin.

[13] CalabrÃ E, Pastorino U (2018). Primary sternal tuberculosis mimicking a lytic bone tumor lesion. Monaldi Arch Chest Dis.

[14] Jambhekar NA, Kulkarni SP, Madur BP, Agarwal S, Rajan MGR (2006). Application of the polymerase chain reaction on formalin-fixed, paraffin-embedded tissue in the recognition of tuberculous osteomyelitis. J Bone Joint Surg Br.

